# Differential responses to ^223^Ra and Alpha-particles exposure in prostate cancer driven by mitotic catastrophe

**DOI:** 10.3389/fonc.2022.877302

**Published:** 2022-07-28

**Authors:** Francisco D. C. Guerra Liberal, Hugo Moreira, Kelly M. Redmond, Joe M. O’Sullivan, Ali H. D. Alshehri, Timothy C. Wright, Victoria L. Dunne, Caoimhghin Campfield, Sandra Biggart, Stephen J. McMahon, Kevin M. Prise

**Affiliations:** ^1^ The Patrick G Johnston Centre for Cancer Research, Queen’s University Belfast, Belfast, United Kingdom; ^2^ Northern Ireland Cancer Centre, Belfast Health and Social Care Trust, Belfast, United Kingdom; ^3^ Department of Radiological Sciences, College of Applied Medical Sciences, Najran University, Najran, Saudi Arabia

**Keywords:** alpha particles, radium-223, mitotic catastrophe, radiation effects, bone metastases

## Abstract

**Introduction:**

Radium-223 (^223^Ra) has been shown to have an overall survival benefit in metastatic castration-resistant prostate cancer (mCRPC) involving bone. Despite its increased clinical usage, relatively little is known regarding the mechanism of action of ^223^Ra at the cellular level.

**Methods:**

We evaluated the effects of ^223^Ra irradiation in a panel of cell lines and then compared them with standard X-ray and external alpha-particle irradiation, with a particular focus on cell survival and DNA damage repair kinetics.

**Results:**

^223^Ra exposures had very high, cell-type-dependent RBE_50%_ ranging from 7 to 15. This was significantly greater than external alpha irradiations (RBE_50%_ from 1.4 to 2.1). These differences were shown to be partially related to the volume of ^223^Ra solution added, independent of the alpha-particle dose rate, suggesting a radiation-independent mechanism of effect. Both external alpha particles and ^223^Ra exposure were associated with delayed DNA repair, with similar kinetics. Additionally, the greater treatment efficacy of ^223^Ra was associated with increased levels of residual DNA damage and cell death by mitotic catastrophe.

**Conclusions:**

These results suggest that ^223^Ra exposure may be associated with greater biological effects than would be expected by direct comparison with a similar dose of external alpha particles, highlighting important challenges for future therapeutic optimization.

## Introduction

The use of the alpha-particle-emitting radionuclide radium-223 in the treatment of prostate cancer bone metastasis has been an active area of research in recent years. Alpha particles are known to be more radiobiologically effective in killing cells in comparison to low linear energy transfer (LET) radiation such as X-rays or β-particles, with an increased relative biological effectiveness (RBE) of approximately 3 (1). Additionally, alpha-particle-irradiated cells also show reduced radioresistance in the absence of oxygen, with an oxygen enhancement ratio (OER) close to 1.0. Such advantageous radiobiological properties of alpha particles demonstrate their potential in clinical radiotherapy. Underpinning these advantages is the fact that alpha particles and X-rays have distinct ionization patterns. X-rays have a low LET and, therefore, deposit a sparse pattern of ionization, randomly depositing energy throughout the nucleus. However, alpha particles have a high LET which results in dense ionizations along the track, resulting in a higher probability of multiple and complex double-strand breaks (DSBs) which are difficult to repair (2).

These distinctive characteristics between low and high LET radiation explain why high LET radiation, especially short-range alpha particles, has been predicted to be the ideal modality to overcome cancer radioresistance.

In recent years, the bone targeting high LET radionuclide radium-223 (^223^Ra) has been shown to prolong survival in castration-resistant prostate cancer patients with bone metastases (3). This has encouraged the use of ^223^Ra in more extensive clinical trials (4). Despite the increased clinical utility of ^223^Ra, little is known regarding its cellular mechanism of action in treatment settings. There is a pressing need to model and quantify the effects of ^223^Ra in preclinical models to optimally design the next generation of trials (5).

^223^Ra is a calcium mimetic and complexes with hydroxyapatite crystals in osteoblastic bone metastases, meaning that ^223^Ra specifically targets areas in the bone with high metabolic turnover (6). ^223^Ra has been approved by the FDA in 2013 and is given to patients in the form of ^223^Ra dichloride (^223^RaCl_2_) injections (Bayer AG, Leverkusen, Germany: Xofigo^®^).

^223^Ra has a physical half-life of 11.4 days, and each ^223^Ra decay results in the emission of four alpha particles. The range of ^223^Ra-emitted alpha particles is less than 70 µm in soft tissue (3). This small penetration range is important as it minimizes the damage to surrounding tissues.

Many questions remain regarding ^223^Ra dosimetry *in vitro* and *in vivo*. In the literature, a broad range of doses required to achieve an *in-vitro* survival fraction of 50% have been reported, ranging from less than 2 mGy (7) to more than 4.1 Gy (8). Additionally, using a high-throughput approach, Yard et al. reported that the required dose for a 50% survival was around 0.2 Gy, and RBE values in the studied cell lines ranged from 4.5 to 12 (9). As different radiation particles and irradiation methods lead to different biological effects, we for the first time compared the effects of ^223^Ra irradiation on different prostate tumor and bone cellular models to X-ray and external ^241^Am alpha irradiation in order to better understand the biological consequences of ^223^Ra treatments.

## Results

### ^223^Ra dosimetry

The absorbed dose in the nucleus of an attached cell from a ^223^Ra decay was calculated using the TOPAS Monte Carlo code, in a simplified geometry of a six-well plate. In our simulations, ^233^Ra and its progeny were in equilibrium, and activity was uniformly distributed in the medium. From these simulations (described in more detail in the methods), the average dose to the nucleus of a complete ^223^Ra decay per milliliter of media in the well was 2.198 × 10^−9^ Gy/decay/ml. Knowing the average dose to the nucleus per ^223^Ra decay and the vial activity at the start of treatment, it was possible to calculate the volume of ^223^Ra to be added to each sample for a desired absorbed dose.

**Table 1** shows the required ^223^Ra activity to deliver different absorbed doses as a function of treatment time.

**Table 1:** Initial ^223^Ra activity (kBq) required per milliliter of media as a function of mean nucleus absorbed dose (Gy) and treatment time (hours); statistical uncertainty is approximately 0.03%.

	0.05 Gy	0.1 Gy	0.25 Gy	0.5 Gy	1 Gy	2 Gy
**6 h**	1.0	2.1	5.3	10.6	21.2	42.4
**24 h**	0.3	0.5	1.4	2.7	5.4	10.8

### Cell survival

**Figure 1:**
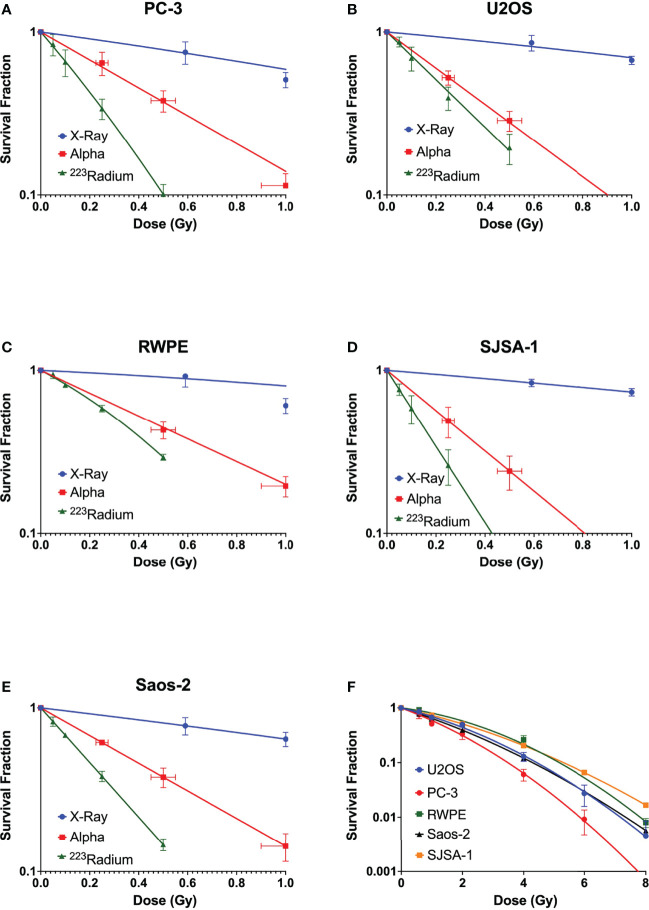
Survival curves of **(A)** PC-3, **(B)** U2OS, **(C)** RWPE, **(D)** SJSA-1, and **(E)** Saos-2 cells obtained after exposure at different doses of X-rays (blue) and external alpha source (red) or exposure to ^223^Ra for 24 h (green). **(F)** Comparison of X-ray survival curves for different cell lines. Data were fit to the linear quadratic model. Points represent the mean of at least three independent experiments with respective standard error.

Clonogenic assays were performed for all five cell lines. All studied cell lines showed the greatest radiosensitivity to ^223^Ra, particularly for PC-3 and SJSA cells (**Figure 1**), as reflected in the relative biological effectiveness for 50% survival (RBE_50%_) values. **Table 2** shows the linear quadratic parameters obtained for the different irradiation setups and the respective RBE_50%_. The sensitivity to ^223^Ra treatment is particularly high for PC-3 cells at 0.5 Gy [Survival Fraction (SF) = 0.09 ± 0.01 and 0.38 ±0.05 for ^223^Ra and external alpha particles, respectively]. The fact that ^223^Ra and external alpha beam results are consistently different from each other for all tested cell lines suggests that there is a common mechanism underlying these differences in response. Some of the differences between the two high LET setups (129 vs. 72 keV/µm, for the alpha source and ^223^Ra, respectively) may be due to differences in doses rates, as ^223^Ra exposures require longer treatments in comparison to external particle irradiation (24 h vs. less than a minute). Interestingly, the most radioresistant cell line in our panel, SJSA, has the highest RBE_50%_ values for external alpha and ^223^Ra exposures.

**Table 2:** Linear quadratic parameters for PC-3, U2OS, RWPE, SJSA-1, and Saos-2 after exposure to different radiation qualities.

Parameters		PC-3	U2OS	RWPE	SJSA-1	Saos-2
**X-rays**	*α* (Gy ^-1^)	0.47 ± 0.03	0.32 ± 0.02	0.16 ± 0.07	0.28 ± 0.05	0.41 ± 0.03
	*β* (Gy ^-2^)	0.05 ± 0.01	0.04 ± 0.01	0.05 ± 0.01	0.03 ± 0.01	0.03 ± 0.01
**Alpha**	*α* (Gy ^-1^)	1.97 ± 0.16	2.55 ± 0.26	1.61 ± 0.10	2.85 ± 0.57	1.94 ± 0.26
	*β* (Gy ^-2^)	~0	~0	~0	~0	~0
**^223^Ra**	*α* (Gy ^-1^)	4.01 ± 0.19	3.53 ± 0.39	1.89 ± 0.15	5.37 ± 0.39	3.84 ± 1.02
	*β* (Gy ^-2^)	1.15 ± 0.26	~0	1.34 ± 0.34	~0	~0
**RBE_50%_ (X-ray/alpha)**		3.6 ± 0.3	6.4 ± 0.6	5.6 ± 0.4	8.5 ± 0.5	4.3 ± 0.1
**RBE_50%_ (X-ray/^223^Ra)**		7.8 ± 0.4	8.8 ± 0.7	7.6 ± 0.6	15.8 ± 0.7	8.3 ± 0.3

### Radiation-induced DNA damage and repair kinetics

Immunofluorescence staining of 53BP1 was employed to detect DNA repair protein foci corresponding to DSBs in three of the five cell lines: one representing normal tissue, RWPE; one representing prostate cancer, PC-3; and one representing bone cancer, U2OS.

**Figure 2:**
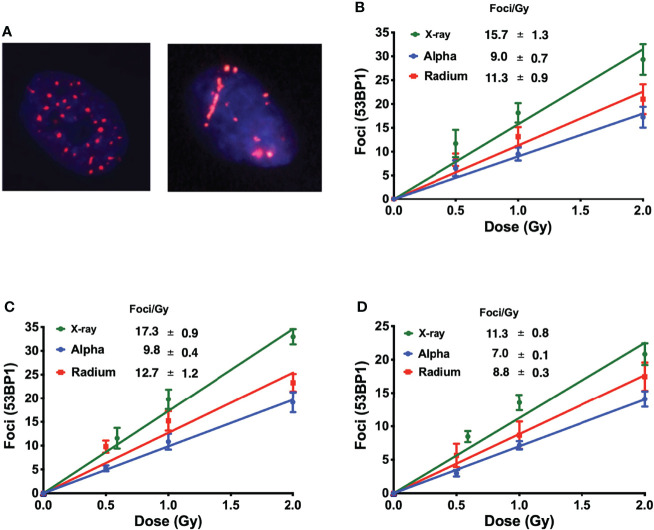
**(A)** Different ionization patterns visualized by immunofluorescence staining for 53BP1 in the nucleus of U2OS cells 1 h after exposure to 1 Gy of X-rays (left) and ^223^Ra (right). The average number of 53BP1 foci per cell induced 1 h after exposure to X-rays or external alpha particles or 24-h exposure to ^223^Ra for **(B)** PC-3, **(C)** U2OS, and **(D)** RWPE. Points represent the mean of three independent experiments and the respective standard error; all values are background-corrected for the average of foci in control samples.

Interestingly, this allows the identification of different ionization patterns of different radiation qualities. X-ray-induced 53BP1 foci are randomly distributed throughout the nucleus, with smaller dimensions and brightness when compared with alpha-particle-induced foci. Alpha-particle-induced foci are bigger and brighter and are in closer proximity to each other following the densely ionizing alpha-particle track (**Figure 2A**).

**Figures 2B–D** show the dose–response of each cell line to different irradiations 1 h after exposure. The DNA damage curves are radiation- and cell-specific. X-ray irradiation showed the highest induction of foci. This is followed by ^223^Ra which induced approximately 25% fewer foci than X-rays, and finally, the external alpha particles induced the lowest number of foci, approximately 40% fewer than X-rays.

**Figure 3:**
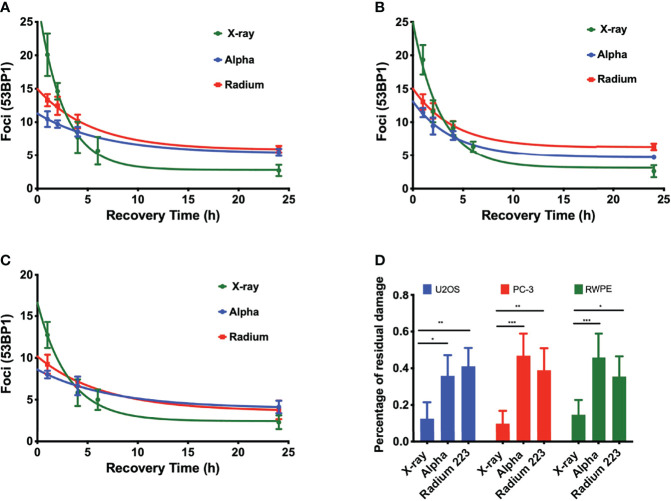
LET-dependent repair kinetics of radiation-induced foci. The repair of alpha-particle-induced foci is slower than those induced by X-rays (green) regardless of whether high LET was delivered by an external alpha source (blue) or 24-h exposure to ^223^Ra (red), for **(A)** PC-3, **(B)** U2OS, and **(C)** RWPE. **(D)** Percentage of residual damage at 24 h after irradiation normalized to the initial yield of damage. Points represent the mean number of foci per cell of three independent experiments and the respective standard error. Data were corrected for the baseline mean foci value and fit to an exponential decay equation. Analysis was performed using t-student method; A significant change when compared to the control group is represented by * (*p* < 0.05; ** (*p* < 0.01); *** (*p* < 0.001); **** (*p* < 0.0001).

DNA damage repair was also evaluated. Samples were irradiated with 1 Gy, fixed, and stained at different time points up to 24 h. Although there is a significantly higher level of DSBs with X-rays, DSB repair is slower with alpha particles (**Figure 3**). This is particularly evident when considering foci recovery percentages. Approximately 85% of X-ray-induced DSBs are repaired after 24 h, but only about 50% of alpha-particle-induced DSBs are repaired in a similar timescale. Moreover, while the initial rate of DSB repair shows an LET dependence, no difference was seen between external alpha and ^223^Ra irradiation (**Table 3**) (*p* ≥ 0.5 for all cell lines).

**Table 3:** Initial rate of DSB repair (k) and DSB repair half-life (T_half_)for different irradiation setups; the half-life is calculated from 0.69/k.

Parameters		PC-3	U2OS	RWPE
**X-rays**	*k* (h ^-1^)	0.39 ± 0.08	0.36 ± 0.06	0.33 ± 0.07
	*T*_half_ (h)	1.75	1.92	2.13
	Fraction residual damage	0.10 ± 0.07	0.12 ± 0.09	0.14 ± 0.08
**Alpha**	*k* (h ^-1^)	0.14 ± 0.09	0.24 ± 0.05	0.14 ± 0.06
	*T*_half_ (h)	4.57	2.77	5.09
	Fraction residual damage	0.47 ± 0.12	0.36 ± 0.11	0.46 ± 0.013
**^223^Ra**	*k* (h ^-1^)	0.18 ± 0.05	0.23 ± 0.06	0.15 ± 0.08
	*T*_half_ (h)	3.81	2.81	4.56
	Fraction residual damage	0.39 ± 0.11	0.41 ± 0.10	0.36 ± 0.11

### ^223^Ra dose rate and solution toxicity

**Figure 4:**
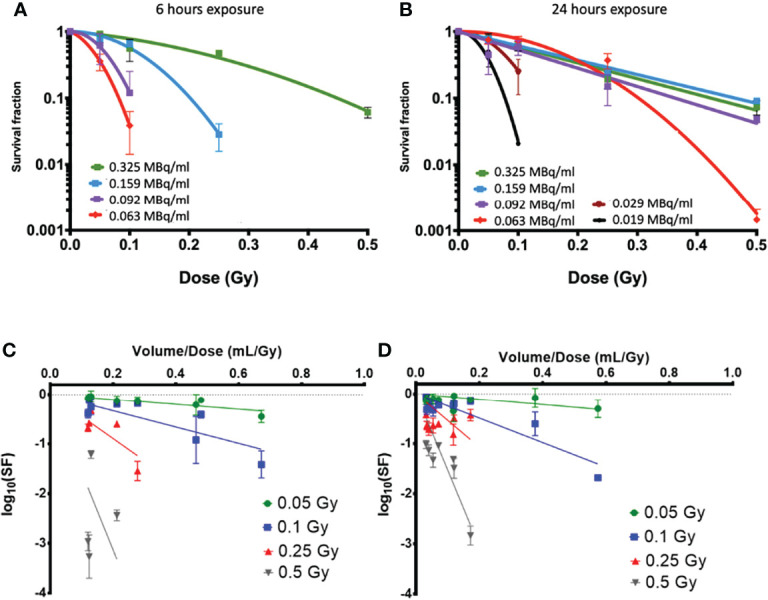
Clonogenic survival data on PC-3 cells exposed to ^223^Ra for **(A)** 6 h and **(B)** 24 h using vials with different activities per milliliter at the time of the experiment [denoted in legend in **(A, B)**]. While all cells were treated with the same activity for a given dose and time, different volumes of ^223^Ra stock solution were needed to achieve a given dose, showing clear differences in survival. Here, the SFs are displayed as logarithm values. This can also be seen as a function of added volume when cells were exposed to alpha-particle doses of 0.05, 0.1, 0.25, and 0.5 Gy from ^223^Ra exposures for **(C)** 6 h and **(D)** 24 h.

**Figures 4A, B** show the clonogenic survival of PC-3 cells exposed to the same dose of ^223^Ra delivered over 6 or 24 h. The results from the two different delivery times are significantly different, showing considerably greater survival for longer exposures. For example, for a 0.5-Gy dose, survival was around 10% for 24-h exposures and less than 0.2% for 6-h exposures. Part of the difference in the result may be attributed to dose rate, as there is greater cell recovery for longer exposures; however, the cell-killing effect of long ^223^Ra exposures is still higher than acute external alpha irradiation.

By using solutions with different initial activity, it can be seen that there is higher toxicity when a greater volume of the radium stock solution is used in the treatment, even when this corresponds to the same dose delivered at the same dose rate. This suggests that there may be some additional effect causing cell killing by the radium solution, independent of radiation dose but instead related to the total volume of solution delivered.

The dose–response in PC-3 cells appears to be consistent for 24-h irradiations with stock activities above 0.09 MBq/ml. However, as the added volume increases, either due to shorter delivery times or reduced initial vial activity, the cells show significantly greater sensitivity.

**Figures 4C, D** show an analysis of the effect of the added volume of stock ^223^Ra solution (*V*) on survival, which is directly related to the vial activity. To deliver the same radiation dose at the same dose rate, as the vial activity decreases, the treatment volume increases. This analysis compares the surviving fraction to the treatment volume/dose ratio (*V*/D_treat_), which is inversely proportional to the stock activity for a given delivery time and dose. For all treatment doses and exposure times, the cell survival decreases when the volume of ^223^Ra added is increased.

### Evaluation of nucleus morphology

Nuclear morphology after exposure to X-rays, external alpha particles, and ^223^Ra was evaluated to understand the mechanism of cell death. **Figure 6** shows the three categories of observed nuclear morphologies: (**A**) normal cell nuclei; (**B**) giant nucleus or arrested cell, defined as when the nucleus has more than 2.5 times the average area of the control nuclei; and (**C**) aberrant nucleus, due to problems in cytokinesis and chromosome segregation after a failed attempt at mitotic division (mitotic catastrophe).

**Figure 5:**
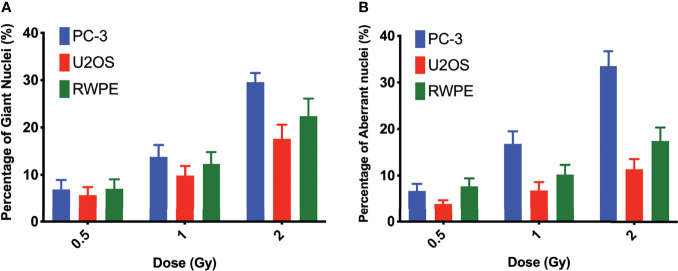
^223^Ra induces mitotic catastrophe in a cell- and dose-dependent manner. Percentage of cells that have **(A)** a giant nucleus 24 h after irradiation or **(B)** an aberrant nucleus 96 h after irradiation as a function of absorbed dose. Data were obtained by microscopic evaluation of cell morphology after DAPI staining of three independent experiments with the respective standard error.

**Figure 6:**
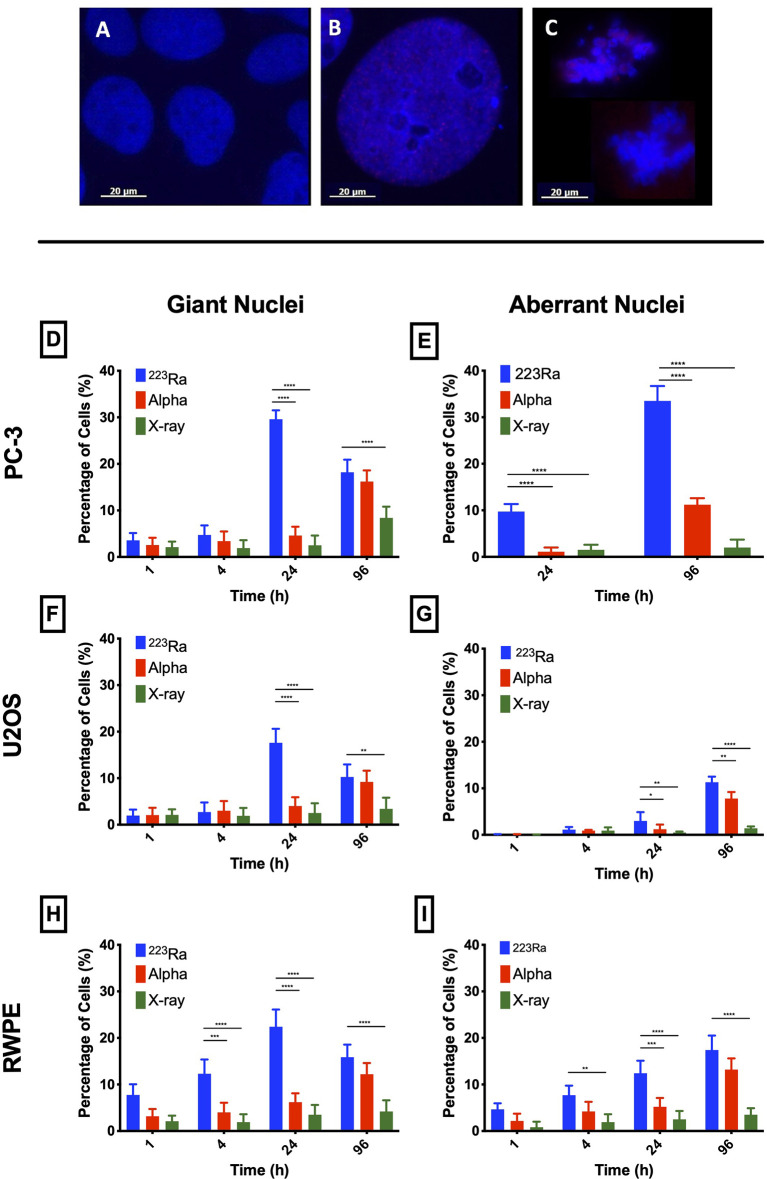
Typical nuclear morphologies observed after 1 Gy of ^223^Ra irradiation in 24 h and fixed at 96 h after exposure; cell nuclei were labeled with DAPI. **(A)** U2OS control cells. **(B)** Morphology of a giant nuclei cell after irradiation. **(C)** Aberrant nucleus representative of cells that undergo mitotic catastrophe after irradiation. **(D*–*I)** Mitotic catastrophe in response to ^223^Ra irradiation is cell-specific. In all cell lines [PC-3 **(D, E)**, U2OS **(F, G)**, and RWPE **(H, I)**], there is a peak of giant nuclei at 24 h after ^223^Ra exposure and a peak for aberrant nuclei at 96 h after 2 Gy of irradiation. Bars represent the mean of three independent experiments and the respective standard error. Analysis was performed using t-student method; A significant change when compared to the control group is represented by * (*p* < 0.05; ** (*p* < 0.01); *** (*p* < 0.001); **** (*p* < 0.0001).

The measurement of the rates of these morphologies in cells exposed to ^223^Ra suggests that mitotic catastrophe following ^223^Ra exposure is cell- and dose-dependent (**Figure 5**). At 24 h, ^223^Ra induces a high percentage of cells with giant nuclei, presumably cells that have arrested in the G2/M phase, in comparison with X-rays and external alpha-particle irradiation (*p* < 0.0001 two-way ANOVA between the cell line and radiation type). At 96 h, ^223^Ra irradiation also induced an elevated percentage of aberrant nuclei (**Figure 6**). These observations suggest that elevated levels of mitotic catastrophe may be the main reason for increased ^223^Ra effectiveness in comparison with external alpha particles.

### Cell cycle profile after irradiation

**Figure 7:**
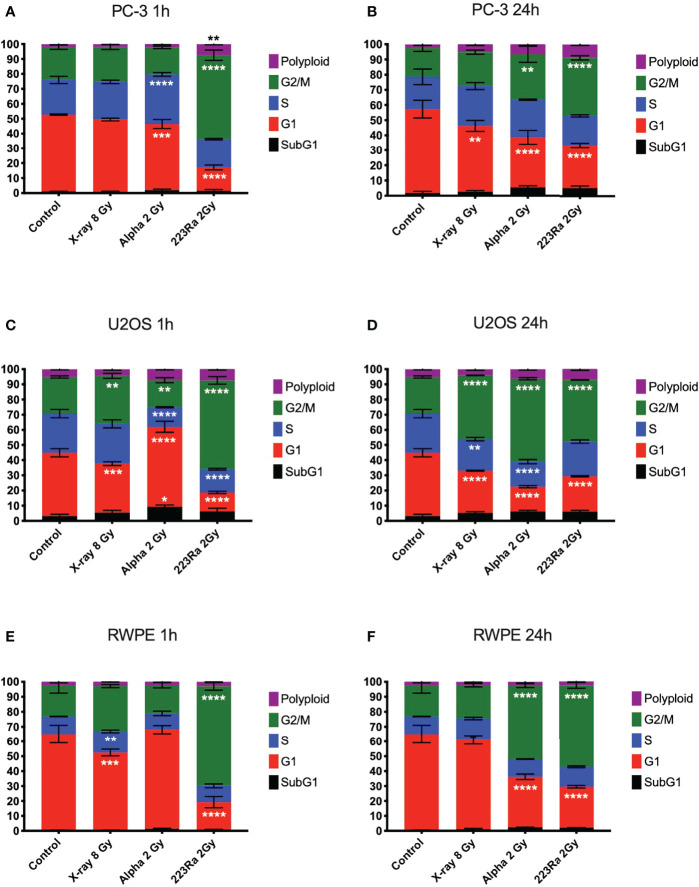
Cell cycle analysis of PC-3, U2OS, and RWPE 1 h **(A, C, E)** or 24 h **(B, D, F)** after treatment with 8 Gy of X-ray, 2 Gy of external alpha particles, or 2 Gy of ^223^Ra delivered in 24 h. Bars represent the mean of two independent experiments and the respective standard error. A significant change when compared to the control group is represented by * (p < 0.05), ** (p < 0.01), *** (p < 0.001), and **** (p < 0.0001).

DNA damage data strongly suggest that alpha particles cause delayed repair and higher levels of residual damage. We investigated the impact of this DNA damage on the cell cycle profile at 1 and 24 h after irradiation. The cell cycle profiles of PC-3, U2OS, and RWPE were determined by flow cytometry using propidium iodine (PI) staining of DNA, plotted in **Figure 7**. The cell cycle distribution of treated cells is substantially different from control samples irrespective of the radiation quality used. However, cells treated with alpha particles show a greater increase in the G2/M phase. The percentage of cells in G2/M increases from 19.1 ± 2.4 for the controls in PC-3 cells to 22.3 ± 1.3, 29.9 ± 4.3, and 38.2 ± 1.4 for 8 Gy of X-ray, 2 Gy of alpha source or 2 Gy of ^223^Ra delivered in 24 h, respectively. Similar trends are seen in the other two cell lines, with significantly greater arrest for higher LET irradiation.

In all three cell models, it is notable that cells exposed to ^223^Ra show a significant arrest in the G2/M phase as soon as 1 h after irradiation, a characteristic not seen with the external alpha-particle irradiation. This is due to the different irradiation conditions, with external alpha irradiation being delivered in less than 2 min. In contrast, ^223^Ra samples are exposed for 24 h, so a significant proportion of cells will have been affected by alpha particles earlier in the exposure, giving time for buildup at the G2/M checkpoint. It is interesting to note that G2/M peaks for the two alpha irradiations are comparable at 24 h after exposure, suggesting that this arrest has a long duration.

### ^223^Ra impact on apoptosis

**Figure 8:**
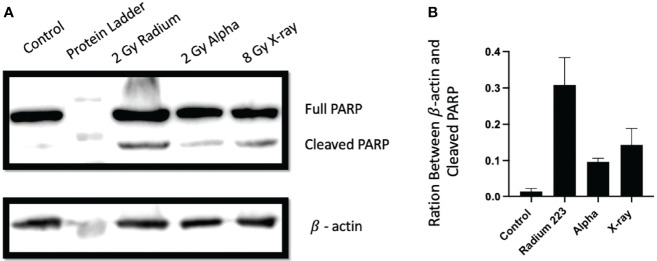
Different exposure conditions induce PARP cleavage in PC-3 24 h after irradiation. **(A)** Western blot demonstrating the protein expression of full and cleaved PARP-1 and β actin at 24 h following irradiation with 8 Gy of X-ray, 2 Gy of ^223^Ra, or2 Gy of external alpha beam. **(B)** The ratio between cleaved PARP-1 and β actin (loading control). Bars represent the mean of two independent experiments and the respective standard error.

To investigate the involvement of apoptotic cell death in cells treated with ^223^Ra, apoptosis was evaluated by assessing the cleavage of PARP-1, a hallmark of apoptosis induction. Overall, all radiation qualities significantly increase the levels of cleaved PARP-1 at 24 h after exposure (**Figure 8**). Furthermore, significant differences are seen between 2 Gy ^223^Ra and external alpha exposures, with ^223^Ra inducing higher amounts of cleaved PARP-1. These differences between high LET irradiation setups may again be related to differences in the irradiation times.

## Discussion

We studied the *in-vitro* radiation effects of ^223^Ra to improve our understanding of its effectiveness and mechanism of action, compared with external alpha or reference X-ray exposures. A better understanding of the effects of radionuclide exposures can help us to develop more efficient clinical treatment schedules and prescriptions. This work has focused on an absorbed dose range that matches with clinical estimates of the mean absorbed dose to metastatic bone tumors after ^223^Ra treatments (0.05–2 Gy) (10–12).

As expected, alpha-particle irradiation is more effective than conventional X-rays with RBE_50%_ of up to 8, in agreement with previously published values (1, 13). Additionally, these results are in agreement with the proposed direct action of high LET radiation on DNA, as reflected with a linear survival fit for external alpha irradiation. Surprisingly, when investigating ^223^Ra, all studied cell lines showed greater sensitivity to ^223^Ra in comparison with external alpha particles (**Figure 1**). Survival measurements across the five cell lines for ^223^Ra in comparison to X-rays and external alpha particles suggest significant diversity in cellular response. Similar cellular and genetic diversity has been previously reported in a panel of 28 cell lines (9).

It is unclear whether the greater efficacy of ^223^Ra is the result of the different dose rates used in the analysis (1.4 mGy/min ^223^Ra vs. 1.59 Gy/min external alpha particles) or by cell-type-specific intracellular uptake of ^223^Ra. For example, intracellular sequestration of ^223^Ra by the iron-storage protein ferritin has been demonstrated (14). In the present study, we were unable to detect any residual activity in the cells after ^223^Ra removal; however, we cannot completely exclude ^223^Ra cellular uptake due to sensitivity limitations. This is a topic of ongoing research (15).

Another explanation for this difference between ^223^Ra and the external alpha source could be a physical phenomenon not simulated in the dosimetric study, such as the settling of ^223^Ra atoms on the bottom of the well during exposure, as dosimetric studies assumed a homogeneous distribution of activity.

It is also possible that the efficacy of ^223^Ra is linked to some non-radioactive constituents of ^223^Ra solution or ^223^Ra decay products, such as heavy metals, which are known to be toxic to cell cultures. Thus, the effect of varying volumes of ^223^Ra solution in PC-3 cells was explored. Volumetric analysis showed that when using stock vials with considerably different activities to deliver the same treatment dose at the same dose rate, there was significantly higher cell killing as vial activity decreased (and, thus, stock solution volume added to the culture medium increased). This was noticeable for all vials with activity at or below 0.55 MBq. For example, the survival of PC-3 cells exposed to 0.1 Gy was 0.61 ±0.09 when vial activity was 1.95 MBq, but 0.02 ±0.01 when vial activity was 0.11 MBq. This shows a 30-fold decrease in survival although the treatments were identical in terms of both dose and dose rate. This volume effect was evident across all samples when survival was analyzed in terms of stock solution volume used, with a clearly decreasing survival with increasing volume.

The DNA damage data presented here are consistent with previous results, showing that alpha particles induce DSBs of higher complexity and these complex DSBs take between two and three times longer to be repaired (**Figure 3** and **Table 3**). The main reason why alpha particles induce more complex DSBs is related to their high LET. For alpha particles, with increasing LET, the dense ionization pattern along their track results in collections of multiple or complex DSBs in close proximity to each other (1, 2). In this work, alpha particles from the external source interacted with the cell nucleus with an average energy of 2.88 ±1.04 MeV and a corresponding LET of 129 keV/µm, and alpha particles from ^223^Ra interacted with the nucleus across a broader range of energies with an average of 5.6 MeV and LET of 72 keV/ µm.

Despite the difference in LET, and in contrast to clonogenic data, immunofluorescence staining failed to a show major difference of DSB induction and kinetics between ^223^Ra and external alpha particles, suggesting that the superior effectiveness of ^223^Ra is not driven by different levels of DNA damage. The small differences found in the detected number of foci between the two alpha-particle sources are most likely due to geometrical considerations in the irradiation setup. In ^223^Ra irradiations, alpha particles are randomly distributed around the cell with a range of incident angles, making the detection of individual foci along the alpha track easier. In contrast, alpha particles from the external source will traverse the cellular nuclei perpendicular to the observational plane, potentially overlapping, making it more challenging to correctly distinguish individual foci within the same track. Similar variations in different microscope setups have been previously reported (2). However, these geometrical considerations can also have biological consequences, as external irradiation will predominantly be traversing the minor axis of the attached cell, with shorter tracks, while with ^223^Ra irradiation, the cells will be irradiated from a wide range of angles that generate much longer tracks through the cell nucleus. As demonstrated previously, longer tracks are typically more biologically effective as they affect multiple chromosomal territories and deposit more dose (16).

One cellular response to radiation-induced DNA damage is cell cycle arrest in order to allow time for DNA repair. A significant G2/M cell phase arrest was observed, in good agreement with previously published data (8, 17, 18). Our data (**Figure 7**) showed a distinct cell cycle profile between X-rays and high LET irradiation in all studied cell lines. Exposure to high LET radiation led to a greater arrest in the G2/M phase at 24 h after irradiation than X-rays, and this arrest was similar between external alpha and ^223^Ra at this time point. However, ^223^Ra exposure was the only irradiation setup to induce a significant G2/M arrest at 1 h after exposure in all cell lines. This is likely due to the long exposure time (24 h), as in this scenario there are some cells that have been affected by the radiation up to almost 25 h before the cell cycle measurements. It is important to note that the G2/M checkpoint is insensitive in situations of a low number of DSBs (<10–20 DSBs), and in low-dose rate scenarios, such as the 24-h ^223^Ra exposures in this study, some cells will progress to mitosis with unrepaired DSBs which may lead to genomic instability or mitotic catastrophe (17).

To evaluate this, changes in nuclear morphology as a response to different irradiations were also investigated. A significant increase of nuclei with more than twice the area of the control cells was observed 24 h after ^223^Ra irradiation. Also, at 96 h, aberrant nuclei with several distinct lobes were observed. We thus concluded that elevated levels of mitotic catastrophe may be the main reason for the increased ^223^Ra effectiveness in comparison with external alpha-particle irradiation, particularly in the PC-3 cell model, that lacks TP53, which plays a major role in cell cycle progression (19, 20). Our results are in agreement with the literature which identifies mitotic catastrophe as one of the major cell death mechanisms induced by high LET radiation, particularly in cell lines that fail to accurately activate p53-dependent DNA damage checkpoints (21–23). Additionally, the data presented here suggests that after exposures to ^223^Ra, theutationn status of DNA damage response genes (*ATM*, *ATR*, *BRCA1*/2, *TP53*, *PRKDC*, *RAD51*, and others) might play an important role in the response of various cell lines to ^223^Ra exposures. Mutations in different DNA damage response proteins and mitotic catastrophe pathways in some cell lines may possibly increase the therapeutic effect of ^223^Ra compared with low LET radiation. Although historically the effectiveness of alpha particles was only associated with direct complex damage to the DNA, there is increasing evidence that shows that alpha particles also affect extranuclear compartments such as the mitochondria and the cellular membrane and are also able to induce a strong bystander effect extending their biological effects beyond the range of alpha particles (24–27). Additionally, bystander effects have been shown to have a large impact on overall effectiveness in scenarios of low dose and low-dose rate exposure. Here, the ^223^Ra low-dose rate exposure could initiate a stronger bystander effect than the same dose of external irradiation; however, this possibility needs to be tested and validated.

## Conclusions

Radium-223, as an alpha-emitting radionuclide, offers several competitive advantages over standard low LET irradiations, due to its high LET and short path length. In this study, we evaluated the biological effects of ^223^Ra and compared them with conventional X-ray and external alpha irradiation, in a range of absorbed doses that are clinically relevant, from 0.05 to 2 Gy. As previously reported, there is significant variability in the absorbed dose to the tumor between different lesions and patients with reported absorbed doses for an injection of ^223^RaCl_2_ ranging from 0.2 to 1.9 Gy (11). It is also widely accepted that there is a heterogeneous dose distribution profile in the tumor with some areas receiving significantly higher doses than others (5).

The clonogenic assay highlighted significant differences in radiosensitivity between external alpha-particle irradiation and internal irradiation with ^223^Ra. These differences were later shown to be partially related to a treatment volume effect of the ^223^Ra solution but were not related to the amount of DNA damage induced by each radiation modality. The main mechanism of action of alpha-particle irradiation is believed to be by direct damage to the DNA, inducing clustered and complex DSBs that present a much greater challenge to cellular repair; however, recent evidence also shows a significant contribution of extranuclear targets and bystander effects. A stronger bystander effect response in ^223^Ra exposure scenarios may be one of the main reasons of this increased efficacy. In future studies, special attention should be given to the mutational status of DNA damage response genes to fully optimize the therapeutic outcome of ^223^Ra irradiation. Here, it was demonstrated that the very low-dose rate of ^223^Ra gives it an advantage in avoiding the G2/M cell cycle arrest and leading to increased levels of mitotic catastrophe in cell models lacking p53/p21-dependent G1 cell cycle arrest.

Furthermore, high LET irradiation conditions can have a profound impact on the main mechanism of cell death, which in the case of ^223^Ra occurs *via* mitotic catastrophe.

Finally, the clinical application of alpha-emitting radionuclides is continually expanding and evolving, from the application of simple radioactive elements such as ^223^Ra to a conjugated carrier molecule (^225^Ac-PSMA or ^227^Th-PSMA) or combination treatments (^223^Ra plus docetaxel). There are many challenges still ahead and more preclinical and clinical research in the field such as this study is strongly advocated. The data presented here characterize the biological response of different cell lines to ^223^Ra and corroborate the greatest efficacy of alpha-particle irradiation mainly due to increased levels of mitotic catastrophe in a low-dose rate exposure scenario. These findings can help to optimize targeted alpha therapy and explore possible combinatory scenarios with different DNA damage repair inhibitors in particular ones with a direct role in the G1/M checkpoint.

## Materials and methods

### Cell culture

PC-3, RWPE, SJSA-1, Saos-2, and U2OS cell lines were obtained from the American Type Culture Collection (ATCC, Manassas, Virginia, USA) Manassas, Virginia, USA. PC-3 and SJSA-1 cells were propagated in RPMI-1640 medium, U2OS cells were propagated in DMEM medium, and Saos-2 were propagated in McCoy’s 5a medium modified, all supplemented with 10% fetal bovine serum and 1% penicillin–streptomycin. RWPE cells were propagated in keratinocyte serum-free medium (K-SFM) supplemented with pituitary extract (BPE) and human recombinant epidermal growth factor (EGF). All cultures were incubated at humidified 37°C in 5% CO_2_.

### Irradiation setup

The X-ray irradiations were performed using an X-RAD 225 radiation source (Precision X-ray Inc., USA) at 225 kVp, 13.3 mA, at a constant dose rate of 0.59 Gy/min.

For external alpha irradiation, Mylar dishes with a thickness of 0.9 µm were placed 2.9 mm from a 50 × 50-mm planar ^241^Am alpha source, with a dose rate of 1.57 Gy/min. Incident average energy at the target cell was 2.88 ± 1.04 MeV with an LET of 129.3 ± 15.2 keV/ µm, as previously described (28).

For ^223^Ra exposures, the recommended culture media for each cell line were supplemented with the activities of ^223^Ra solution from 0 to 5 kBq/ml and left to incubate for 6 or 24 h. Based on all alpha-particle decays from the ^223^Ra cascade, the average energy of an emitted alpha particle is 6.67 MeV with an initial LET of 72 keV/µm. After the exposure, the treatment medium was removed, and the wells were washed three times with PBS.

The access to ^223^Ra vials resulted from a collaboration with the Northern Ireland Cancer Centre, which kindly donated to research spare ^223^Ra vials that were not used during the clinical treatments. Each ^223^Ra vial contains 6.6 MBq ^22^Ra dicloride in 6 ml of saline solution, at the reference date printed in the label by manufacture. The other ingredients are 6.3 mg/ml of sodium chloride USP, 7.2 mg/ml of sodium citrate USP, 0.2 mg/ml of hydrochloric acid USP, and water USP for injection. It is important to note that different vials had different activities when used in this work due to variations in pickup time, which means that different volumes of the ^223^Ra solution have to be used to achieve the target activity for different experiments; however, the Xofigo volume was always lower than 25 µl per ml of medium, and cells were exposed to a total volume of 2 ml. In all experiments, samples were treated with a saline solution that contained all the elements of the Xofigo solution except for ^223^Ra which was added to the cells in a volume equal to the Xofigo solution added to the highest dose to act as a control for the known components of the Xofigo solution.

### Dosimetry

The absorbed dose in the cells and the hit distribution from the alpha particles of all progenies from ^223^Ra were calculated using the TOPAS Monte Carlo, Oakland, California, USA simulation toolkit (version 3.0), run on an Apple Mac (2012 – Intel i5 3.2 GHz) (29). TOPAS is a user-friendly dosimetry simulation for research and clinical physicists. TOPAS was previously validated for cellular and subcellular dosimetry (30–32).

**Figure 9:**
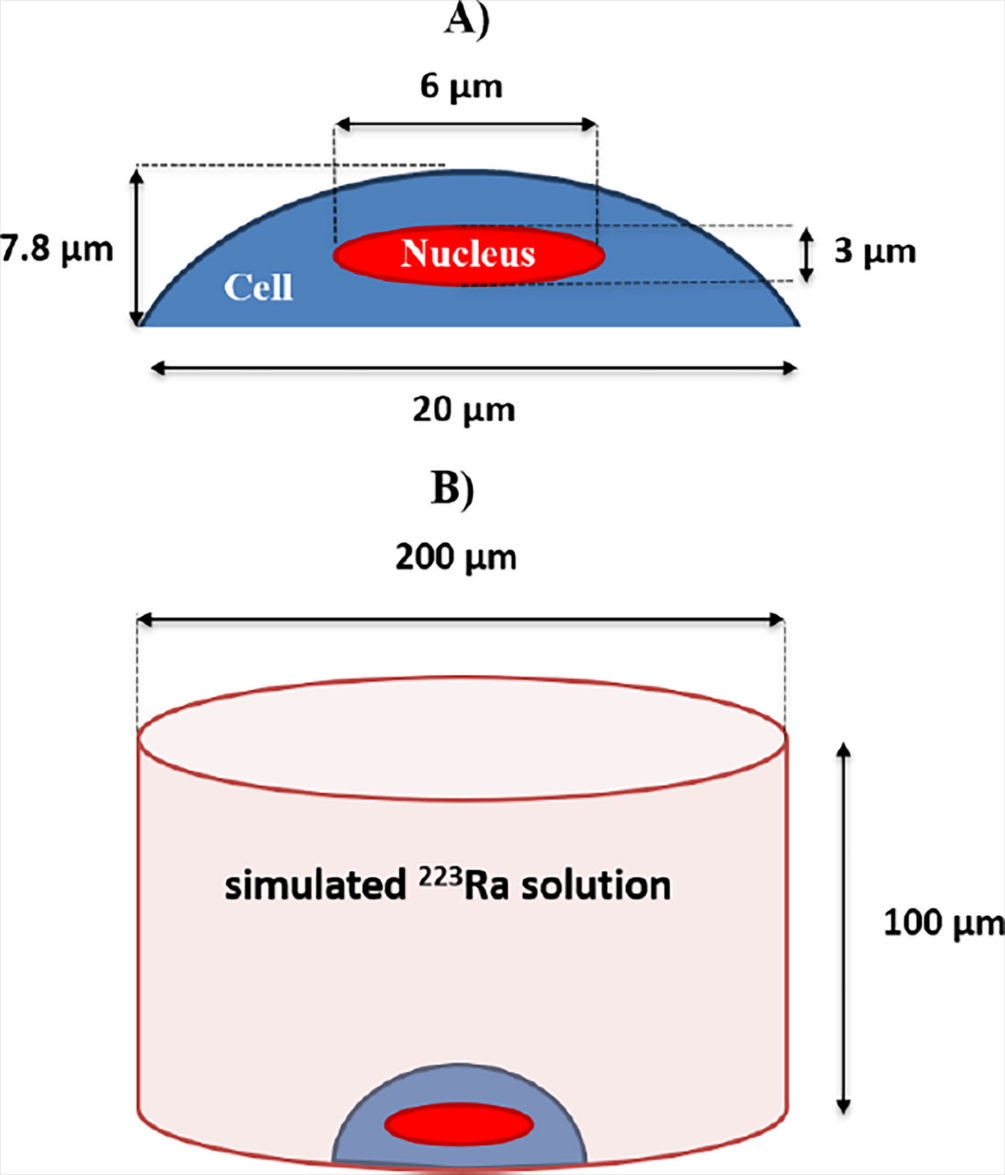
**(A)** The simulated cell as a semi-ellipsoid with 20-μm-side diameters and 7.8-μm thickness. The nucleus is simulated as a full ellipsoid with 6-μm-side diameters and 3-μm thickness, centered at the middle of the cell. **(B)** The simulated cylindrical volume of a treatment solution with alpha-particle emissions that resulted from ^223^Ra decays. A simulated geometry of a cell with its nucleus is placed at the bottom of the simulated well. The simulated well geometry has a 200-μm base diameter, having a 100-μm height.

Doses from beta and gamma radiations were neglected, as they represented less than 4.7% of the emitted energy. A semi-ellipsoid cell geometry (20 µm diameter and 7.8 µm thickness) with a centered ellipsoid nucleus (6 µm diameter and 3 µm thickness), representative of typical cellular dimensions, was simulated and attached to a small section of a well of a cell culture plate (**Figure 9**).

A uniform distribution of ^223^Ra within the simulated solution was assumed. Therefore, all alpha particles were isotropically emitted from random positions inside the simulated media volume, with the restriction that emissions only occurred outside the cell geometry. A total of five independent runs with 100 million alpha-particle cascade decays from ^223^Ra were simulated, incorporating all the alpha-particle emissions from the ^223^Ra decay cascade with the following energies: first cascade emission—5.87 MeV, probability 100%; second cascade emission—6.82 MeV, probability 100%; third cascade emission—7.38 MeV, probability 100%; and fourth cascade emission—6.62 MeV, probability 99.7% or 7.45 MeV, 0.3% probability. For each simulated decay, the dose to the nucleus was scored and averaged to predict the average dose per decay. The resulting dose in the nucleus was 2.198 (± 0.0007) × 10^−9^ Gy/decay/ml.

With information regarding dose deposition to the nucleus per ^223^Ra decay, the number of decays per 1 Bq of ^223^Ra solution for a desired exposure time, and the planned delivered dose, it is possible to calculate the necessary ^223^Ra activity to be added to the cells per milliliter of medium (**Table 1**). From the required ^223^Ra activity at the time of the experiment, it is possible to calculate the volume of ^223^Ra solution to be added to each sample.

### Clonogenic survival assay

The colony formation assay was carried out according to published methods (33). Cells were seeded into six-well plates (Sarstedt AG & Co., Germany) with a cell density depending on the cell line and absorbed dose. On the following day, the cells were irradiated with doses of up to 8 Gy of X-rays or exposed to different activities of ^223^Ra for 6 or 24 h to deliver radiation doses from 0 to 0.5 Gy. After irradiation, cells were then incubated for 10 days. The colonies were stained with crystal violet solution and were manually counted, with a colony defined as consisting of at least 50 cells. From these counts, plating efficiency and survival were calculated. The survival fraction was determined by the number of colonies formed after treatment divided by the number of cells seeded, corrected for the PE of unirradiated cells.

Alternatively, for external alpha-particle beam exposures, cells were irradiated in Mylar dishes with doses up to 2 Gy. After alpha-particle irradiation, cells were trypsinized, counted, and reseeded onto six-well plates, following the same conditions and experimental steps as the other plates.

Data were fit to the linear quadratic equation [ SF=*e* ^−(*αD*+*βD* ^2^)^ ] using non-linear regression. The different irradiation setups were then compared for each cell line to the X-ray response by calculating the relative biological effectiveness (RBE) for SF = 50%.

### DNA damage assay

Following irradiation, cells were fixed in 50:50 methanol–acetone solution and permeabilized (0.5% of Triton X-100 in PBS) at predetermined time points before being blocked in blocking buffer (5% FBS and 0.1% Triton X-100 in PBS) and stained with 53BP1 primary antibody (1:5,000) (NB100-304, Novus Biologicals, USA) for 1 h before being washed three times and stained with Alexa Flour 568 goat anti-rabbit IgG secondary antibody (1:2,000) (A21429, Life Technologies, USA) in the dark for 1 h. Following staining, the cells were washed three times and mounted onto microscope slides using the Prolong Gold antifade reagent with DAPI (P36930, Invitrogen, USA). Foci were manually counted from the whole nucleus of 50 randomly selected cells on each sample with a Zeiss Axiovert 200 microscope (Carl Zeiss), using a ×63 objective. Data are presented as the mean values of foci per cell and the respective standard error of three independent experiments. All presented data were corrected for baseline foci values of control samples, that is, baseline foci numbers for unirradiated cells were subtracted from the treated samples. The baseline values for the cells were 3.1 ± 0.8 for U2OS, 2.8 ± 0.5 for PC-3, 1.4 ± 0.3 for RWPE, 2.0 ± 0.4 for SJSA-1, and 3.9 ± 1.0 for Saos-2. For repair kinetic analysis, foci data were then fit with an exponential decay in GraphPad Prism 7, *N* = (*N*_0_ – *Plateau*) * *e^–kt^* + *Plateau*), where *N*_0_ represents the initial number of 53BP1 foci, *Plateau* represents the residual damage, and *k* is the rate of DSB repair.

### Cell cycle profile analysis

Cells were irradiated with 8 Gy of X-rays or 2 Gy of high LET radiation (either external alpha or ^223^Ra). Cells were harvested 1 or 24 h after irradiation before being fixed in 100% ice-cold ethanol and left at 4°C overnight. The samples were then centrifuged, the excess ethanol was removed, and then cell pellets were resuspended in PBS and centrifuged again before resuspending in 500 μ l of PI/RNase A. The samples were incubated at 37°C for 30 min before being analyzed in a BD Accuri C6 Plus Flow Cytometer (BD Biosciences, USA), and 10,000 flow cytometer events were collected and analyzed per sample. Quantification was carried out using the BD Accuri C6 Plus Analysis software.

### Western blotting

Following irradiation with different radiation qualities, cells were harvested 24 h after treatment and proteins extracted according to published methods (34). A 40- μ g total protein sample was loaded onto a 10% SDS-PAGE gel, and after electrophoresis, the proteins were blotted on a nitrocellulose membrane (Life Technologies, USA). The membranes were blocked with 5% non-fat dairy milk in PBS-Tween (0.1% Tween-20 in PBS) and incubated overnight at 4°C with primary antibody [PARP #9542 (Cell Signaling, USA) at a dilution of 1:1,000 in 5% non-fat milk in PBS]. The anti-β-actin (Cell Signaling, USA) antibody was used as a housekeeping control at a dilution of 1:5,000. After washing with PBS-Tween, the membranes were incubated in their secondary anti-rabbit and anti-mouse horseradish peroxidase-conjugated antibodies diluted at 1:2,000 at room temperature for 1 h. The membranes were then washed and developed with Luminata Crescendo Western HRP substrate (Millipore, USA) using the GBox Imager by Syngene (Cambridge, UK).

### Nuclear morphology analysis

After irradiation, cells were fixed in 50:50 methanol–acetone solution and stained with DAPI and then observed with a fluorescence microscope. From each sample, 200 randomly selected cells were imaged and their morphologic characteristics recorded using the ImageJ (version 1.8.0_172) analysis particle function (35). Cells containing a nuclei area bigger than 2.5 times the mean area of control samples were scored as positive for G2/M arrest. Cells containing nuclei with two or more distinct lobes or aberrant nucleus morphology were scored as positive for mitotic catastrophe.

### Statistical analysis

All experiments were performed in triplicate. Unpaired Student’s *t*-test and one-way ANOVA were used for statistical evaluation. All statistics and graph plotting used GraphPad Prism 7.0 (GraphPad, USA).

## Data availability statement

The original contributions presented in the study are included in the article/supplementary material. Further inquiries can be directed to the corresponding author.

## Author contributions

FL: experimental design, data generation, data analysis, and manuscript preparation and review. HM: experimental design, data generation, and data analysis. KR, VD, TW, and AA: experimental design and data generation. CC and SB: experimental design and reagent provision. JO’S: project supervision, funding acquisition, and manuscript preparation and review. SM and KP: experimental design, project supervision, funding acquisition, and manuscript preparation and review. All authors contributed to the article and approved the submitted version.

## Funding

The authors gratefully acknowledge the support of the Movember/Prostate Cancer UK Centre of Excellence (CEO13_2-004) and the Research and Development Division of the Public Health Agency of Northern Ireland (COM/4965/14). We also acknowledge the support of Fundação para a Ciência e Tecnologia (FCT-MCTES), Radiation Biology and Biophysics Doctoral Training Programme (RaBBIT, PD/00193/2012); UID/Multi/04378/2013 (UCIBIO); and UID/FIS/00068/2013 (CEFITEC). The following scholarship grants were also acknowledged: number SFTH/BD/52534/2014 to HM and SFTH/BD/114448/2016 to FG. SM was supported by UKRI Future Leaders Fellowship MR/T021721/1. AA was supported by the Ministry of Higher Education in Saudi Arabia through the Saudi Arabian Cultural Bureau (SACB) in London. TW was supported by a Northern Ireland Department of the Economy studentship. VD was supported by the LFT Charitable Trust.

## Conflict of interest

JO’S has received honoraria as part of the speakers’ bureau and the advisory board of Bayer and has received institutional research funding from Bayer. KP has received speaker honoraria from Bayer.

The remaining authors declare that the research was conducted in the absence of any commercial or financial relationships that could be construed as a potential conflict of interest.

## Publisher’s note

All claims expressed in this article are solely those of the authors and do not necessarily represent those of their affiliated organizations, or those of the publisher, the editors and the reviewers. Any product that may be evaluated in this article, or claim that may be made by its manufacturer, is not guaranteed or endorsed by the publisher.
